# Infection risk in Rheumatoid Arthritis and Spondyloarthropathy patients under treatment with DMARDs, Corticosteroids and TNF-α antagonists

**DOI:** 10.1186/1479-5876-12-77

**Published:** 2014-03-22

**Authors:** Valentina Germano, Maria Sofia Cattaruzza, John Osborn, Aurora Tarantino, Roberta Di Rosa, Simonetta Salemi, Raffaele D’Amelio

**Affiliations:** 1 Department of Clinical and Molecular Medicine, “Sapienza” University of Rome, S. Andrea University Hospital, Via di Grottarossa 1039, 00189 Rome, Italy; 2 Department of Public Health and Infectious Diseases, “Sapienza” University of Rome, P.le Aldo Moro 5, 00185 Rome, Italy; 3 Current address: Italian Air Force, Logistic Command, Health Service, Viale dell’Università 4, 00185 Rome, Italy

**Keywords:** Rheumatoid Arthritis, Spondyloarthropathy, Infection risk, anti-TNFα, DMARDs, Corticosteroids

## Abstract

**Background:**

Infections which complicate rheumatic diseases such as Rheumatoid Arthritis (RA) and Spondyloarthropathy (SpA) (Psoriatic Arthritis [PA] and Ankylosing Spondylitis [AS]), may cause significant morbidity and mortality. However, among the studies on the incidence rate (IR) of infections in such patients, very few have involved controls and the results have been controversial, probably due to methodological difficulties.

To estimate infection rates in RA and SpA patients under disease-modifying anti-rheumatic drugs (DMARDs), corticosteroids (CS) and tumor necrosis factor (TNF)α antagonists, alone or combined, a single-centre retrospective observational cohort study has been performed.

**Patients and methods:**

Incidence rates/100 patient-years of any infections were evaluated in RA and SpA outpatients observed in the period November 1, 2003 through December 31, 2009 and stratified according to therapy. Infection incidence rate ratios (IRR) were calculated using Poisson regression models which adjusted for demographic/clinical characteristics of the patients.

**Results:**

Three hundred and thirtyone infections [318 (96.1%) non-serious and 13 (3.9%) serious] have been registered among 176 of the 341 patients (52%). The IR/100 patient-years of all infections was 36.3 ranging from 12.4 (DMARDs + CS) to 62.7 (anti-TNFα + CS). The most frequent infection site was respiratory tract, and bacteria were responsible for three quarters of all infections. In the multivariate analysis, adding anti-TNFα to DMARDs doubled the IRR compared to DMARDs alone, anti-TNFα + CS significantly tripled it, whereas anti-TNFα + CS + DMARDs only increased the risk 2.5 times. The degree of disease activity was strongly and significantly associated with the infection risk (severe or moderate versus mild, IRR = 4). Female sex was significantly associated with increased infection risk, while duration of disease and anti-influenza vaccination were protective, the latter even for cutaneous/soft-tissue (mainly herpetic) infections.

**Conclusion:**

The combination anti-TNFα with CS was found to be the most pro-infective treatment, whereas DMARDs alone were relatively safe. Physicians, therefore, should be aware that there may be an increased risk of infection when using anti-TNFα and CS therapy together. Anti-influenza vaccination appears to provide broad protection, adding evidence to support its use in these patients, and deserves further study.

## 

Infections which complicate rheumatic diseases such as Spondiloarthropathy (SpA) (Psoriatic Arthritis [PA] and Ankylosing Spondylitis [AS]) and Rheumatoid Arthritis (RA), may cause significant morbidity and mortality [1-3]. However, among the studies on the incidence rate (IR) of infections in such patients, very few have involved controls and the results have been controversial, probably due to methodological differences.

A Dutch group found no significant difference between RA patients and controls regarding the IR of infections [4]. In contrast, other authors have noted an increased infection risk [5-7]; in particular, Doran et al. [7] in 609 US RA patients found a doubled risk of developing an objectively confirmed infection compared with a sample of 609 age- and sex-matched controls without RA. Contradictory results are probably, at least in part, a consequence of the different events studied, including overall, non-serious, serious or opportunistic infections, different patient groups or different statistical pmeters [8]. Moreover, the high statistical power obtained from large patient populations is frequently counterbalanced by possible bias linked to the scarce availability of detailed clinical information [9,10], which may eventually compromise the outcome. Finally, also the type of study, randomized controlled trial or observational study, may make a difference, particularly in relation to the patient selection criteria. Although, to the best of our knowledge, studies comparing the infection risk between RA and SpA patients are lacking, the risk in the second patient group seems to be low [11].

More recently, with the introduction of biologics in therapy, infection susceptibility has increased. Consequently, some studies, have drawn attention not only to the disease itself, but also to immune suppression induced by corticosteroids (CS), non biological disease-modifying anti-rheumatic drugs (DMARDs) and biologics.

Data on infection risk generally show that DMARDs (mainly methotrexate [MTX]), are relatively safe, whereas there is little debate on the capacity of CS to increase infection susceptibility [12]. Doran et al. analyzing RA patients, mainly treated with DMARDs and CS, did not find an increased infection risk associated with DMARDs, whereas in a multivariate analysis they identified CS as a predictor of infections [13]. Lacaille et al. [9] demonstrated an increased risk of mild and serious infections in RA patients taking CS, but no increase for DMARDs. Partially in contrast, Bernatsky et al., in RA patients found the highest risk of serious infections with CS and immunosuppressive DMARDs [14]. Although several studies have documented the effectiveness of anti-tumor necrosis factor (TNF)α, conflicting results on the possible increase in the number and/or severity of infections have also been reported. In placebo-controlled trials evaluating each one of three TNFα blockers for RA patients, the rate of any infections did not exceed the rate in the placebo group [15-17], while data from a meta-analysis suggest an increased risk for serious infections [18]. Indeed, data from the British Biologics Register estimated a 2-fold increased risk of serious infections [19] and a recent review [20] reported a 4-fold increased risk of serious bacterial infections in RA patients. In the analysis of the RABBIT German Registry, the risk of serious infections was directly related to the dose of CS, especially when associated with TNFα.

The aim of this retrospective observational cohort study was to evaluate the impact of DMARDs, CS and the three TNFα antagonists licensed in Italy between 2001 and 2005, in mono or combination therapy, on non-serious and serious infections in RA and SpA patients.

## Patients and methods

All the RA and SpA (PA and AS) outpatients observed at the Immuno-rheumatology Division of the S. Andrea University Hospital, Rome, during the period November 2003-December 2009 were evaluated and IRs/100 patient-years of non-serious and serious infections were calculated. RA, PA and AS were diagnosed on the basis of the recommendations of the 1987 American College of Rheumatology [21], Moll and Wright [22] and modified New York criteria [23], respectively. Patients were checked at 3-month intervals and stratified according to the type of therapy: DMARDs alone, DMARDs + CS, anti-TNFα alone, anti-TNFα + CS, anti-TNFα + DMARDs and anti-TNFα + DMARDs + CS. The anti-TNFα therapy was introduced following accepted guidelines [Disease Activity Score (DAS) on 44 joints > 3.7 or Bath Ankylosing Spondylitis Disease Activity Index (BASDAI) > 4] in the absence of contraindications. In particular, patients were excluded in the presence of any active infection after screening with the tuberculin skin test (TST), chest radiograph and hepatitis B (HBV) and C (HCV) viral markers. TST positive patients underwent microbiological and further radiological assessment, in order to exclude active overt disease. In patients without active disease, isoniazid was administered for at least 2 months before starting biological therapy and continued for a total of 6–9 months [24,25]. HBV active and inactive carriers underwent an anti-viral therapy or prophylaxis, respectively, whereas occult carriers were treated with biologics. In this case, viral markers including DNA levels and hepatic function were monitored throughout therapy [26,27]. HCV-antibody positivity was not considered a cause of exclusion, but, in the case of HCV viremia, specific anti-viral treatment before and quantitative viral evaluation and serum alanine aminotransferase monitoring after biological therapy was suggested [27,28].

The Ethics Committee of the S. Andrea University Hospital approved the study (approval number 132/2012). All infections occurring during treatment were noted either at planned or at patient solicited medical checks for any new onset clinical event. Patients were actively, but only orally, solicited to immediately notify any possible adverse event occurring during the treatment, especially infections. These infections were actively looked for and diagnosed on the basis of clinical assessment and response to anti-infective treatment. Only the infections reported in the medical records collected during outpatient visits were considered. Moreover, they were classified according to demonstrated (by imaging and/or microbiological analyses) or presumed etiology (bacterial, viral and/or fungal) and site (respiratory, uro-genital, gastrointestinal, skin and soft tissue, bone and joints). Among respiratory infections, influenza-like-illness (ILI) episodes have been identified on the basis of the following clinical pmeters: “acute respiratory tract infection and fever ≥ 38°C accompanied by systemic or respiratory symptoms” [29]. Moreover infections were defined as either serious when life-threatening, requiring hospitalization and/or intravenous anti-infective treatment, or non-serious when only requiring a physician visit and/or use of not intravenous anti-infective medications. Comorbidities (diabetes mellitus, leukopenia, chronic obstructive pulmonary disease [COPD]) and smoking status, were considered. Disease activity was classified as mild, moderate and severe when DAS was <2.4, 2.4-3.7, >3.7 and BASDAI 1–3, 4–7 and 8–10, respectively. Anti-influenza and anti-pneumococcal vaccinations, only administered to patients with mild/moderate disease activity in order to prevent any flare up, were recorded.

### Statistical analysis

Proportions were calculated for the demographic and clinical characteristics of all patients. For important clinical characteristics, IRs, defined as the number of observed events (infections)/100 patient-years of follow-up, were calculated, to estimate the risk of infection in the different groups. Also, Incidence Rate Ratios (IRR) and their statistical significance were obtained from Poisson regression analysis. In crude analyses, statistical differences were evaluated by χ^2^ tests and Fisher’s exact test. Variables considered as potential confounders or independent predictors of developing an infection, were included in the Poisson regression model according to univariate results and clinical considerations. Adjusted robust IRR and confidence intervals were calculated using an extra-Poisson variation regression model. Compared to the usual Poisson regression model, this gives the same estimates but wider confidence intervals (through greater standard errors). All analyses were performed using Stata (Stata College Station, TX version 8).

## Results

Demographic and clinical characteristics of patients and infection rates are reported in Table 1. The patients provided a total of 911.8 patient-years of follow-up, with a median follow-up time of 26.04 months (interquartile range 13.77 to 47.28), in particular 21.4% of patients were followed for 12 months or less and 23.2% for more than 48 months. One-hundred and seventy-six (52%) patients had at least 1 infection, the total infections being 331 (96.1% non-serious versus 3.9% serious; mean infections/patient 1.9, range 1–8).

**Table 1:** Demographic and clinical characteristics of patients and crude infection rates/100 patient-years according to the type of treatment

	**DMARDs**	**DMARDs + CS**	**Anti-TNFα**	**Anti-TNFα + CS**	**Anti-TNFα + DMARDs**	**Anti-TNFα + DMARDs + CS**	**All patients**
**Patients N**	30	82	24	42	42	121	341
**Gender**							
Male	8	15	12	13	11	34	93
Female	22	67	12	29	31	87	248
**Age**							
<50	9	15	12	17	14	38	105
50-59	9	22	3	7	8	29	78
60-69	5	22	6	4	13	34	84
≥70	7	23	3	14	7	20	74
**Type of disease**							
RA	9	64	8	29	23	90	223
SpA	21	18	16	13	19	31	118
**Disease duration**							
<5 years	21	41	7	6	14	28	117
5-9 years	2	29	3	18	11	43	106
≥10	7	12	14	18	17	50	118
**Vaccination for influenza**							
Yes	5	24	4	18	17	57	125
No	25	58	20	24	25	64	216
**Comorbidities and smoking**							
Yes	13	24	17	16	21	55	146
No	17	58	7	26	21	66	195
**All infections N**	7	29	16	70	43	166	331
**Serious infections N**	0	0	1	2	2	8	13
**Patient-years**	49.2	233.5	52.7	111.6	103.9	360.9	911.8
**Infections /100**	14.2	12.4	30.4	62.7	41.4	46.0	36.3
**Patient-years**							
**p value**	Reference group	0.721	0.09	0.00001	0.0039	0.0003	-

CS: Corticosteroids; DMARDs: Disease Modifying Anti-Rheumatic Drugs; TNF: Tumour Necrosis Factor.

The infection rate in the first 6 months of treatment was 3.8 per 100 patient-months, and in the subsequent semesters was 3.3, 3.3, 2.7 and 3.0. The chi squared test for the comparison of the semesters and the test for trend are not statistically significant.

The difference between the rates for males and females was statistically significant, females being at increased risk. There was a tendency for the rate to decrease with increasing age-group, but this was not statistically significant. The rates for the two types of diseases, RA and SpA, were similar. The rate increased with disease activity, especially for the “moderate” and “severe” categories; however the very low rate observed for the “mild” group was based on few cases. The risk showed a decreasing trend with disease duration, but this was not statistically significant. Vaccination against influenza and the absence of comorbidities tended to reduce the infection rates, but these were not statistically significant at the 5% level.

The sites and types of non-serious and serious infections are reported in Table 2. One male patient under anti-TNFα + CS therapy died from pneumonia of presumed viral etiology, whereas no other mortality or permanent sequelae were observed as a consequence of infections. Moreover, 16/229 (7%) anti-TNFα-treated patients were positive at TST screening, without radiological and/or microbiological tubercular findings. All of them underwent prophylactic treatment two months before starting biologics and no tuberculosis reactivation was observed in the follow-up. Finally, no HBV surface antigen positivity was found, whereas HBV core antibodies were positive in 12% of patients; all were investigated for HBV viremia with negative results, and thus they were considered potential occult HBV carriers [25]. No HBV reactivation was observed in these patients during the follow-up. Three observed HCV positive cases, one of whom also positive for viremia, were treated with biologics (the patient with viremia only after a pegylated interferon and ribavirin three-month treatment course), without viral reactivation.

**Table 2:** Site and type of non-serious and serious infections

	**Non-serious**	**Serious**	**Total**
	**N (%)**	**N (%)**	**N (%)**
	**318 (96.1)**	**13 (3.9)**	**331 (100)**
**Site of infection**			
Respiratory	120 (36.3)	7 (2.1)	127 (38.4)
Uro-genital	111 (33.5)	0	111 (33.5)
Skin and soft tissue	66 (20.0)	4 (1.2)	70 (21.2)
Gastrointestinal	21 (6.3)	0	21 (6.3)
Bone	0	2 (0.6)	2 (0.6)
Type of infection*			
Bacterial*	**236 (71.3)**	**12 (3.6)**	**248 (74.9)**
*Escherichia coli*	40 (12.1)	0	40 (12.1)
*Staphylococcus aureus*	4 (1.2)	4 (1.2)	8 (2.4)
*Proteus mirabilis*	3 (0.9)	0	3 (0.9)
*Klebsiella pneumoniae*	2	0	2
*Serratia marcescens*	1	0	1
*Yersinia spp*	1	0	1
*Chlamydia trachomatis*	1	0	1
Viral*	**61(18.4)**	**1**	**62 (18.7)**
Herpes simplex	40 (12.1)	0	40 (12.1)
VZ virus	8 (2.4)	0	8 (2.4)
Fungal*	**21 (7)**	**0**	**21 (7)**
*Candida albicans*	5 (24)	0	5 (24)
*Malassezia furfur*	4 (19)	0	4 (19)

*It was not possible to identify micro-organism for all infections.

The IR/100 patient-years for all infections was 36.3 (95% CI from 32.4 to 40.3), but it was higher in the presence of anti-TNFα therapy than with DMARDs alone (the control or reference group) or with CS, as reported in Table 1. When anti-TNFα therapy was combined, especially with CS, the infection risk significantly increased. DMARDs (90% MTX) and CS (prednisone) were administered at low-dose (10–15 mg/weekly and <10 mg/daily, respectively). The IR/100 patient-years for serious infections was 1.4.

The adjusted results obtained from the Poisson regression are reported in Table 3. These results are broadly similar to the crude comparisons. However, the adjusted analysis implied a significant increase for females, a significant decrease in the risk with increased duration of the disease and influenza vaccination was significantly protective towards infections, in particular towards skin and soft tissue infections as shown in Table 4.

**Table 3:** Poisson regression incidence rate ratios

	**Crude IRR**	**Adjusted IRR**	**95% CI**		**p**
Gender female vs male	1.26	1.37	1.01	1.86	0.042
Age (effect per 10 years)	0.88	0.95	0.86	1.05	0.302
Disease duration 5–9 vs <5 years	0.92	0.67	0.48	0.93	0.016
Disease duration 10+ vs <5 years	0.82	0.61	0.45	0.83	0.002
DMARDs + CS vs DMARDs^^^	0.87	0.76	0.34	1.68	0.493
Anti-TNFα vs DMARDs^^^	2.14	1.57	0.55	4.51	0.400
Anti-TNFα + CS vs DMARDs^^^	4.42	3.60	1.58	8.20	0.002
Anti-TNFα + DMARDs vs DMARDs^^^	2.92	2.11	0.95	4.71	0.067
Anti-TNFα + DMARDs + CS vs DMARDs^^^	3.24	2.54	1.15	5.59	0.021
Presence of comorbidities* and smoking	1.22	1.07	0.85	1.37	0.554
Anti-influenza Vaccine	0.84	0.73	0.57	0.94	0.016
Moderate Disease Activity vs Mild at baseline	5.95	4.12	1.24	13.68	0.021
Severe Disease Activity vs Mild at baseline	11.29	4.28	1.21	15.08	0.024
SpA vs RA	0.91	0.96	0.67	1.38	0.817

*presence of at least one of the following comorbidities: COPD, diabetes and leukopenia.

^^^DMARDs = control group.

COPD: Chronic Obstructive Pulmonary Disease; CS: Corticosteroids; DMARDs: Disease Modifying Anti-Rheumatic Drugs; RA: Rheumatoid Arthritis; SpA: Spondyloarthropathies; TNF: Tumour Necrosis Factor. IRR:Incidence Rate Ratio, CI: confidence interval.

**Table 4:** Effect of influenza vaccination on respiratory, uro-genital, skin/bone/soft tissue and gastro-enteric infection risk

**Site of infections**	**Influenza vaccination**	**Number of infections**	**Patient-years of follow-up**	**Rate**	**p**
Respiratory	No	74	165.57	0.447	
	Yes	53	128.26	0.413	0.666
Uro-genital	No	58	117.92	0.492	
	Yes	53	113.65	0.466	0.781
Skin/Bone/Soft tissue	No	52	61.91	0.840	
	Yes	20	85.49	0.234	0.0001
Gastro-enteric	No	13	28.09	0.463	
	Yes	8	29.84	0.268	0.229

## Discussion

In this study, an IR/100 patient-years of 36.3 was observed for all infections in 341 RA and SpA patients, being 34.9 for non-serious and 1.4 for serious infections. These results are similar to those reported from the CORRONA Register on a larger RA US patient population (32/100 patient-years) [30], while other studies have reported higher (48.2) [31] and lower rates (22.6, 28.3 and 6.8 for treatment with Etanercept, Infliximab and non biological DMARDs, respectively) [32]. For serious infections, the rate observed in this study is generally lower than those observed in other studies, excepting that in the CORRONA Register, where the IR was 0.8/100 patient-years for hospitalized infections [30]. The IRs/100 patient-years for serious infections are, in fact, higher in the German Biologics Register (6.4, 6.2 and 2.3, for treatment with Etanercept, Infliximab and non biological DMARDs, respectively) [32] and in the British Register (5.3 and 4.1 for biologics and non biological DMARDs, respectively) [19]. In a recently published multicenter retrospective cohort study, RA patients had IRs 8.2 versus 7.8 for biologics versus non biological DMARDs, and for psoriasis/spondyloarthropathy the rates were 5.4 versus 5.4 respectively [10]. Moreover, an Italian RA population study [33] reported an IR 3.6.

The percentage of patients infected among those treated with DMARDs and biologics is seldom reported, but varies from 13% [32], through 23% [30] and 34.5% [31], to 51% [34]. Among the cases described here, it is 176/341, 52% (146 [83%] of whom were treated with biologics and 111 [63%] of them also with CS in addition to biologics). Despite the difficulty of providing an interpretation of this variability, as proposed by Salliot et al. [31], the different percentages of patients on CS and/or anti-TNFα therapies may in part account for the observed variation. Indeed, in the current study, the proportions of patients treated with anti-TNFα (229/341 [67%]) or with CS (245/341 [72%]) (anti-TNFα and CS associated in 163/341 [48%]) are higher than in other studies (45% and 24% respectively in Au et al. [30]), but lower, for CS, than in the German Biologics Register [32], where it was 82%.

Infections were mainly (> 90%) respiratory, uro-genital or cutaneous/soft tissue, as found by others [31,32]. Skin and soft tissue infections were considered important in patients under anti-TNFα therapy even in 2006 [20,35], probably as a consequence of the TNFα role in cutaneous immunity [36]. TNFα, in fact, is a key cytokine responsible for cutaneous endothelial activation and thereby recruitment of inflammatory cells to the skin. Moreover, it is also important in the mobilization of cutaneous antigen-presenting cells (Langerhans cells) from the epidermis to draining lymph nodes [37,38] The virulence of *Staphylococcus aureus* is confirmed, having been isolated in over 30% of the serious infections. Contrary to Favalli et al. [33], in this study there were no cases of active tuberculosis, probably because the patients were enrolled after 2001 [39], when sensitivity to possible tubercular reactivation in anti-TNFα-treated patients became very high. The percentage of HBV core antibody positivity is lightly higher than, but not significantly different from, that reported by Caporali et al. (12% versus 9%) [40]. Lack of HCV reactivation in the three treated patients is in line with the literature [28,41].

IRs/100 patient-years in the different patient groups stratified according to treatment type range from 12.4 (DMARDs + CS), to 14.2 (DMARDs), to 30.4 (anti-TNFα alone), to 41.4 (anti-TNFα + DMARDs), to 46.0 (anti-TNFα + DMARDs + CS) to 62.7 (anti-TNFα + CS). Thus, CS behave as immunosuppressants when associated with anti-TNFα, but are less influential when combined with DMARDs, whereas biologics seem to be associated with enhanced infection risk. In contrast to other authors [10,42,43], we did not observe a temporary increase of infection risk in the first period after start of immunosuppressive therapy.

The multivariate analysis shows that the type of disease (RA or SpA) does not significantly affect the infection risk (IRR 0.96), therefore the two patient groups have been considered as a single population. This may be probably due to a sort of “balancing” effect of immunosuppressive therapy on the different infection risk of RA and SpA *per se*. Comorbidities have not been found to significantly affect the infection risk (IRR 1.07), but the confidence interval is wide (0.85-1.37). It would be interesting to investigate the effects of the single comorbidities and combinations of them, but the sample size is too small for an analysis of this type. However, in females, the infection risk is increased by more than one third, as already observed by Lacaille et al. [9] and by Au et al. [30]. Female gender as a pro-infective element may be linked to the impact of uro-genital infections which are more common in females [44]. Disease duration is generally considered a risk factor for infections [19,45], whereas in this study, surprisingly, it was protective. The crude effects are not statistically significant, but disease duration is very likely to be confounded with other factors, for example age, the presence of comorbidities and disease activity, and this may explain the increased and statistically significant effect observed in the adjusted analysis. Indeed, protection may be linked to the high proportion (> 90%, data not shown) of patients with mild-moderate disease activity during the follow-up (patients with ≥ 2 years of treatment) as a consequence of treatment effectiveness. Despite the limited evidence regarding the impact of the disease activity on the susceptibility for infections [46], recently Au et al. found that higher disease activity was associated with a higher probability of developing hospitalized infections [30]. In the current study, baseline disease activity is closely associated with infection risk, with an IRR of 4.3 and 4.1 for severe and moderate versus mild disease activity, respectively. Chronic inflammatory diseases and infections are both interwoven with disease activity. High disease activity, in fact, is the expression of chronic inflammatory state, which in turn may suppress the immune system [47], thus making the development of infectious diseases easier. Conversely, infections may strongly stimulate the immune system, thus enabling, through inflammation, the underlying disease to reactivate. Both processes are able to increase the level of the indices for disease activity.

The most interesting results come from treatment type. Anti-TNFα alone increases the IRR and when combined with CS more than triples it compared with DMARDs alone. Moreover, DMARDs seem to provide protection against infection in patients taking biologics, CS and DMARDs together, compared with biologics and CS alone, the IRR being 2.5, instead of 3.6. In agreement with recent studies, which have identified a significant risk for non-serious [7-9,48] and serious [8-14,46,49-52] infections for even low-dose CS (< 10 mg/day of prednisone) the current study confirms that they are pro-infective and thus likely immunosuppressive, especially if combined with anti-TNFα. Moreover, the association CS/anti-TNFα therapy has been recently described as strong predictor of serious infections in RA [46,52,53]. Biologics were instead associated with a net increased infection risk, mainly when administered with CS. DMARDs (mainly MTX) appear to be safer regarding infection risk, with an IR/100 patient-years (12.9) compble to that (12.87) observed by Doran et al. [7] in non RA controls. The apparent lack of MTX pro-infective action, or rather its possible protective effect, has already been reported [9,19]. This may be partly related to its anti-methylenetetrahydrofolate reductase activity, which is able to inhibit bacterial [54] and viral [55,56] proliferation. The important increase in the risk of infection associated with the combined treatment CS + anti-TNFα may instead be because CS, in addition to the induction of apoptosis on T lymphocytes [57], are able to reduce cytokine production [57]. This includes TNFα which appears to be the most sensitive [58]. Anti-TNFα therapy is only able to inactivate TNFα, which is increased in RA patients, without effect on its synthesis. This may, instead, be inhibited by CS [59]. Anti-TNFα agents, on the other hand, may reverse the TNFα-induced CS resistance in RA, by restoring the TNFα-inhibited glucocorticoid receptor function [60,61]. The CS/anti-TNFα combination, therefore, synergizes in lowering TNFα levels through different and independent mechanisms, with consequent enhanced anti-inflammatory effect, but at the expense of a raised infection risk [52]. Physicians, therefore, should be aware that there may be an increased risk of infection, when using CS and anti-TNFα therapy together. However, this result is not obtained from a randomized clinical trial. There may be residual confounding due to factors which have not been included in this study, which could explain the association between increased risk of infection and the combination of CS and anti-TNFα therapy. Thus, the topic deservers further studies.

As found by Schneeweiss et al., patients treated with anti-TNFα were more likely to have received at least one influenza vaccination [50]. Moreover, having been vaccinated at least once against influenza was found to be significantly protective towards the overall risk of infection in the multivariate analysis. This observation appears important, but there are caveats. First, lack of solid evidence of influenza vaccine efficacy in immunosuppressed populations [62,63]; second, the impossibility to establish a correlation between vaccine administration and protection from virologically confirmed influenza in the same year; third, the finding that vaccination is protective against all infections, with varying IRR, but significantly (p < 0.001) only for skin/bone/soft tissue infections. However, although vaccination may be confounded with unrelated factors, such as compliance with long term medication, or the preferential recruitment of patients with mild/moderate disease activity among the vaccinees, its effect should be reduced or eliminated in a regression model which takes account of these factors. On the contrary, our results imply that the protective effect of vaccination is increased and remains statistically significant after adjustment for these variables. The very low number of ILI episodes (14 patients, 2 of whom vaccinated and 12 non vaccinated) may explain the lack of statistical significance for ILI. Moreover, the high statistical significance found for skin/bone/soft tissue infections, (the majority of which were herpetic [> 55%]), may perhaps be associated with a specific protective action. This observation is in agreement with Miller JB who in 1979 reported that flu vaccine reduced discomfort produced by both, influenza and herpes infections [64]. Furthermore, it has recently been demonstrated that a significantly higher anti-influenza response is induced by a plasmid influenza nucleoprotein DNA vaccine combined with herpes simplex virus (HSV) viral protein 22 gene. This may imply a high level of cross-induced anti-influenza protection by HSV antigen [65].

This study has potential limitations because it is a retrospective analysis of observed clinical results. There was no *a priori* consideration of sample size and power because the patients included were those observed in the Immuno-rheumatology Division during the selected time period. However, it can be calculated that, although the number of patients is relatively low, the study would have had a power of more than 99% to detect the observed difference between treatment with DMARDs + CS and treatment with anti-TNFα + CS significantly at the 5% level. The relatively small sample size may lead to some real associations not being detected and confidence intervals may be so wide as to include clinically important values. Moreover, the low sample size may be offset by the study being single-centre, thus eliminating inter-centre variability, which may prevent under-ascertainment and/or misclassification of infectious events or other clinical information.

Another potential source of bias is inherent in the design of the study. The patients included in the study were those who attended the Immuno-reumathology Clinic during a fixed period of time; that is, they were prevalent cases (in the period) not incident. This may have lead to a higher probability of inclusion for patients with long duration of disease, that is patients with a better prognosis. Given that the time interval for inclusion was relatively long, six years, we believe this bias is unlikely to have had a great effect on the conclusions regarding the incidence of infections in the treatment groups.

A further limitation of the study is the lack of matching among groups exposed to different therapeutic protocols (Table 1), but this is a direct and unavoidable consequence of the study type. Moreover, the evaluation of infection risk for RA and SpA patients together, has been performed following the non-significant comparison between RA and SpA in the multivariate regression. It could be hypothesized that immunosuppressive therapy may have balanced the infection risk between the two different pathological conditions. Finally, comorbidities have been analyzed as a whole and not singularly because of the small number of cases in each; this may miss some important effects and may account for discrepancies observed with some literature data.

## Conclusion

In conclusion, this study suggests a high pro-infective potential of the combination of anti-TNFα agents and CS, and provides a biological interpretation of the synergy. According to EULAR [66] recommendations, CS should be tapered as soon as possible. Indeed, our results suggest that, even though CS may delay radiographic joint damage [67], they should be used carefully with biologics. The search for personalized treatment options should be driven by these considerations, trying to maintain with DMARDs the remission phases induced by biologics, as indicated by EULAR [66]. The observed association between influenza vaccination and skin, bone, soft tissue infections has never been described before, to the best of our knowledge, is interesting, and deserves further study. Thus this may represent a further rationale for the already recommended administration of influenza vaccine to these patients [68-70]. However, larger prospective studies analyzing the role of CS, non biological DMARDs and single biologics on infection risk should be planned and implemented, in order to give a definitive answer to the question of the safety for the immune system of these treatments and their cost effectiveness.

## Abbreviations

AS: Ankylosing spondylitis; BASDAI: Bath ankylosing spondylitis disease activity index; COPD: Chronic obstructive pulmonary disease; CS: Corticosteroids; DAS: Disease activity score; DMARDs: Disease-modifying anti-rheumatic drugs; HBV: Hepatitis B virus; HCV: Hepatitis C virus; HSV: Herpes simplex virus; ILI: Influenza-like-illness; IR: Incidence rate; IRR: Incidence rate ratios; MTX: Methotrexate; PA: Psoriatic arthritis; RA: Rheumatoid arthritis; SpA: Spondyloarthropathies; TNFα: Tumor necrosis factor; TST: Tuberculin skin test.

## Competing interests

The authors declare that they have no competing interests.

## Authors’ contributions

All authors have made substantial contribution to conception and design, acquisition, analysis or interpretation of data. They were involved in drafting the article or writing it critically for important intellectual content. All authors read and approved the final manuscript.
